# Folding and persistence times of intramolecular G-quadruplexes transiently embedded in a DNA duplex

**DOI:** 10.1093/nar/gkab306

**Published:** 2021-05-01

**Authors:** Phong Lan Thao Tran, Martin Rieu, Samar Hodeib, Alexandra Joubert, Jimmy Ouellet, Patrizia Alberti, Anthony Bugaut, Jean-François Allemand, Jean-Baptiste Boulé, Vincent Croquette

**Affiliations:** Structure et Instabilité des Génomes, Museum National d’Histoire Naturelle, INSERM, CNRS, Alliance Sorbonne Université, 75005 Paris, France; Laboratoire de physique de L’École Normale Supérieure de Paris, CNRS, ENS, Université PSL, Sorbonne Université, Université de Paris, 75005 Paris, France; Institut de Biologie de l’École Normale Supérieure (IBENS), Ecole Normale Supérieure, CNRS, INSERM, Université PSL, 75005 Paris, France; Laboratoire de physique de L’École Normale Supérieure de Paris, CNRS, ENS, Université PSL, Sorbonne Université, Université de Paris, 75005 Paris, France; Institut de Biologie de l’École Normale Supérieure (IBENS), Ecole Normale Supérieure, CNRS, INSERM, Université PSL, 75005 Paris, France; Structure et Instabilité des Génomes, Museum National d’Histoire Naturelle, INSERM, CNRS, Alliance Sorbonne Université, 75005 Paris, France; Depixus SAS, 3-5 Impasse Reille, 75014 Paris, France; Structure et Instabilité des Génomes, Museum National d’Histoire Naturelle, INSERM, CNRS, Alliance Sorbonne Université, 75005 Paris, France; Structure et Instabilité des Génomes, Museum National d’Histoire Naturelle, INSERM, CNRS, Alliance Sorbonne Université, 75005 Paris, France; Laboratoire de physique de L’École Normale Supérieure de Paris, CNRS, ENS, Université PSL, Sorbonne Université, Université de Paris, 75005 Paris, France; Institut de Biologie de l’École Normale Supérieure (IBENS), Ecole Normale Supérieure, CNRS, INSERM, Université PSL, 75005 Paris, France; Structure et Instabilité des Génomes, Museum National d’Histoire Naturelle, INSERM, CNRS, Alliance Sorbonne Université, 75005 Paris, France; Laboratoire de physique de L’École Normale Supérieure de Paris, CNRS, ENS, Université PSL, Sorbonne Université, Université de Paris, 75005 Paris, France; Institut de Biologie de l’École Normale Supérieure (IBENS), Ecole Normale Supérieure, CNRS, INSERM, Université PSL, 75005 Paris, France; ESPCI Paris, Université PSL, 75005 Paris, France

## Abstract

G-quadruplex (G4) DNA structures have emerged as important regulatory elements during DNA metabolic transactions. While many *in vitro* studies have focused on the kinetics of G4 formation within DNA single-strands, G4 are found *in vivo* in double-stranded DNA regions, where their formation is challenged by the complementary strand. Since the energy of hybridization of Watson-Crick structures dominates the energy of G4 folding, this competition should play a critical role on G4 persistence. To address this, we designed a single-molecule assay allowing to measure G4 folding and persistence times in the presence of the complementary strand. We quantified both folding and unfolding rates of biologically relevant G4 sequences, such as the cMYC and cKIT oncogene promoters, human telomeres and an avian replication origin. We confirmed that G4s are found much more stable in tested replication origin and promoters than in human telomere repeats. In addition, we characterized how G4 dynamics was affected by G4 ligands and showed that both folding rate and persistence time increased. Our assay opens new perspectives for the measurement of G4 dynamics in double-stranded DNA mimicking a replication fork, which is important to understand their role in DNA replication and gene regulation at a mechanistic level.

## Graphical Abstract

**Graphical Abstract:**
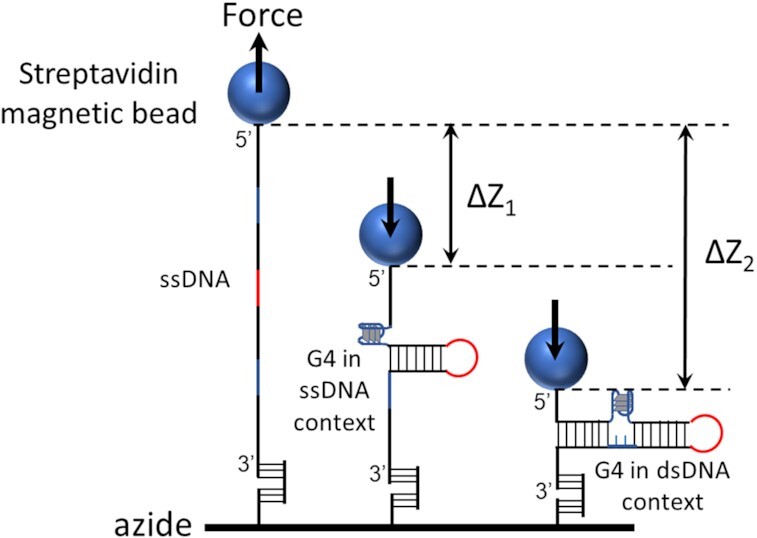
A single-molecule assay allowing to measure G-quadruplex folding and persistence times in the presence of the complementary strand using magnetic-tweezers.

## INTRODUCTION

Nucleic acid sequences with four or more stretches of multiple guanines (G*n*N*x*G*n*N*x*G*n*N*x*G*n*; *n* ≥ 2, *x* ≤ 7) can form non-canonical four-stranded structures called G-quadruplexes (G4s). These structures are assembled by stacking of coplanar guanine’s quartets (G-quartets) that are stabilized by monovalent cations, such as K^+^ or Na^+^. G4 structures are well described at the structural level. Depending on their sequence, they can adopt different folding patterns and can form from the same (intramolecular) or distinct (intermolecular) nucleic acid strands (1–3). Potential G4-forming motifs are widespread across the genomes of a large spectrum of organisms from bacteria to mammals (4–8), and the formation of stable G4 structures *in vivo* has been linked to replication (9–11), oncogene expression (12–14), translation (15–17), DNA repair (18,19) telomere maintenance (20) or epigenetic marking of a DNA locus (21–23). Questions around the physiological conditions conducive to their formation and their biological roles have made G4 structures the focus of extensive *in vitro* and *in vivo* studies over the last two decades.

Single-stranded (ss) nucleic acid sequences, such as the telomeric 3′-overhang or RNAs, are prone to G4 formation with no challenge from a complementary strand. However, G4 forming sequences are allegedly also found in double-stranded (ds) DNA regions, such as promoters (24), minisatellites (25) or replication origins (10). These structures, if stable and unprocessed by helicases (26), may hinder the replication fork and transcription bubble progression, causing fork pausing or promoting DNA breakage (25,27,28). Despite causing potential roadblock for molecular motors, current wisdom is that their formation has been harnessed by evolution to encode structural information within DNA, at the expense of evolving protein motors able to remove the stable structures challenging replication (26,29).

Their potential biological effect would require their persistence post replication in a context where they are embedded in a dsDNA bubble. Persistence of G4 structures within dsDNA is largely unknown, but may depend on their sequence, the genomic context and/or the presence of a bound protein (30–36). Convincing evidence of G4 formation *in vivo* has indeed been reported near several transcription start sites (37) or near-replication origins where G4 structures could participate in transient fork pausing or loading of proteins (10,11,38). All these roles suggest that once formed these structures have a significant persistence time in a dsDNA context.

To date, our knowledge of the conditions of G4 formation is largely inferred from *in vitro* biophysical studies of different model G4-forming single-stranded sequences. These approaches have contributed largely to our understanding of G4 stability and structural diversity, but are limited in providing a complete biologically relevant picture of the dynamics of G4 structures at the molecular level.

Folding/unfolding of several G4s (such as human telomeric or cMYC promoter sequences) have been studied in recent years using magnetic or optical tweezers (39–43). Most of these studies address the dynamics of G4 in a ssDNA context. In a non-replicative dsDNA context, the presence of a complementary strand causes a competition between the G4 and the duplex structure. At the thermodynamic level, the energy of hybridization of a Watson-Crick duplex containing a G-rich DNA sequence is much larger (∼50 kcal/mol) than the energy of G4 folding (∼4–8 kcal/mol) (1). Therefore, the competition with a complementarity strand should play a major role in the formation and persistence of G4 structures *in vivo*, as recently pinpointed by Chalikian *et al.* (35). Two reports using single-molecule FRET or optical tweezers have shown that G4 can readily compete with the reannealing of dsDNA *in vitro* (44,45). However, the persistence time of the G4 structure embedded in duplex DNA remains largely unknown.

Here, we developed an original and label-free single-molecule setup using magnetic tweezers in order to gain insights into this aspect of G4 dynamics. This setup allows measuring formation and persistence of G4 structures in an alternating context between ssDNA and dsDNA, by opening and closing periodically single molecule dsDNA hairpins. This label-free assay allowed us to measure the folding time (*T*_f_) of G4 structures as well as their persistence time (*T*_p_) under a defined force cycle. Using this assay, we compared four well-studied model G4 sequences from different genomic regions, namely human telomeric, human promoters cMYC and cKIT, and a replication origin G4 from the chicken genome. Our experiments show that these sequences form G4 structures as expected but show great variation in their folding and persistence times in ways that are not fully described while considering thermal melting parameters from bulk experiments.

Finally, this system also allowed us to assess the effect of a chemical G4 ligand (360B) (46) and an anti-G4 single-chain antibody (BG4) (47,48) on G4 dynamics. We observed that both molecules favor G4 structure formation by reducing the apparent folding time and increasing the persistence time of the G4. This observation confirms the basic but important idea that some G4s revealed experimentally by such G4 binders (specifically BG4 and 360B) may not form significantly in the absence of ligand.

## MATERIALS AND METHODS

### Oligonucleotides and G4 binders

The oligonucleotides used in this study are described in Supplementary Tables S1 and S2. All oligonucleotides were purchased from Eurogentec (Seraing, Belgium). Lyophilized oligonucleotides were resuspended in distilled water and stored at –20°C. The G4 ligand 360B was synthesized in the laboratory by Patrick Mailliet. 360B is similar to the G4 ligand 360A, but the iodine counter ions in 360A are replaced by sulphonates (46,49) (Supplementary Figure S12A). This modification improves solubility of the compound in aqueous buffers, without affecting its specificity and affinity toward G4 (Patrick Mailliet and Jean-François Riou, personal communication). The BG4 single chain antibody was purified and provided by the laboratory of Prof. Kevin D. Raney (50).

### Experimental setup

The PicoTwist^®^ magnetic tweezers instrument used to manipulate individual DNA hairpins tethered between magnetic beads and a coverslip was described previously (51). Briefly, the magnetic bead was held by a force due to a vertical magnetic field gradient generated by a pair of permanent magnets. Controlling the distance of the magnets from the sample surface allows applying a precisely calibrated variable force on the samples. The movement of these magnets was achieved with the help of DC-motor, which allowed us to exert a force with sub-picoNewton accuracy. The force was calculated from the Brownian fluctuations of the tethered bead (51). The DNA substrate used in the single-molecule studies consisted of a 1.1 kb hairpin containing the G4 forming sequence at ∼640 nucleotides from the ssDNA–dsDNA junction of the molecule. The hairpin also contains a 5′-tri-biotinylated ssDNA tail and a 3′ end with 43-nucleotide-long ssDNA tail (Supplementary Figure S1 and S2). We attached the hairpin substrate at the 5′ end to a streptavidin-coated magnetic bead and at the 3′ end to a glass cover slip through annealing with ssDNA oligonucleotides covalently attached on the surface by click chemistry. Relevant oligonucleotides for hairpin synthesis are described in Supplementary Table S1. To image the beads, a CMOS camera at the image plane of the objective was used to track the position of the magnetic beads in three dimensions with nanometer resolution. Thus, tracking the *z*-axis fluctuations of the tethered beads enabled us to monitor the change in extension of the tethered DNA hairpin in real time with an accuracy of about 5 nm. The extension of the DNA changed in accordance with the applied force or by the presence of hybridized oligonucleotides or a G4 structure. Between 50 and 100 beads were tracked simultaneously in real time in order to obtain statistically significant measurements of single molecule events.

### Experimental conditions for single molecule G4 structure manipulation

All experiments were performed at 25°C in 10 mM Tris–HCl (pH 7.5), with 100 mM KCl or 100 mM LiCl, as indicated in the text and figure legends. Potassium ions (K^+^) strongly stabilize G4s by coordination with the carbonyl oxygens of guanines in the central cavity. To confirm that the detected structures were G4s, control experiments were systematically carried out in 100 mM LiCl. Lithium ions (Li^+^) are too small to fit in the G4 central channel and, hence, stabilizes G4 to a much lesser extent than potassium (52). The experimental system was based on manipulating multiple dsDNA hairpins tethered to a glass slide on one end and to magnetic beads on the other end. Time resolved experiments were started with injection of 10 nM of a 7-base-long oligonucleotide (‘blocking oligonucleotide’) complementary to the loop of the hairpin. In the case of fast folding and persistent G4 structures formed by ori β^*A*^, the blocking oligonucleotide concentration was lowered to 5 nM. In experiments using G4 ligands, ligands and blocking oligonucleotides were injected concomitantly.

### Data collection and analysis

The image of the bead displayed diffraction rings that were used to estimate its 3D position as previously described (53). From position fluctuations of the bead, both the mean elongation of the molecule and the force applied to it could be deduced (54). The *z*-axis fluctuations were acquired at 16 Hz. A typical experimental acquisition spanned almost 24 h, corresponding to ∼2000 open/close cycles. Bead position at each force (2, 7 or 20 pN) were automatically recorded and analyzed using a software developed in house (Supplementary Figure S3–S5). The experimental mean folding time ($\overline{T_{\rm f}}$) of each G4 sequence was inferred by dividing the whole time spent in the unfolded state (the cumulative time of oligonucleotide blockage before a structure is observed), summed over all beads and over the entire acquisition, by the number of times a G4 structure was formed ($\overline{T_{\rm f}}=\Sigma$ oligo blocking time spent in unfolded state/total number of folded G4). This average is the best estimator for the parameter of a single exponential law if one considers G4 folding and unfolding as Poisson processes (see Supplementary Figure S7 for explanations on calculations of the parameters and their independence on experiment time). The mean unfolding time, or persistence time ($\overline{T_{\rm p}}$), was similarly obtained by dividing the whole time spent in the folded state (≥*T*_hold_ = 15 s) by the number of times a G4 is unfolded ($\overline{T_{\rm p}}=\Sigma$ time spent in folded state (≥15 s)/total number of unfolded G4).

The relative error made on these estimations is $\sqrt{N}$, where *N* is the number of observed events (respectively unfolding and folding events). All results presented below were computed from at least 100 independent G4 events. When two successive cycles showed a blockage at the position of the G4, we assumed that it was due to the same structure and that no unfolding and refolding took place during one single hairpin opening phase. If the typical $\overline{T_{\rm f}}$ was much larger than *T*_*c*_ (cycle time), the probability that a G4 unfolded and then refolded during the opening phase was so low that this assumption does not bias the estimation of $\overline{T_{\rm f}}$ and $\overline{T_{\rm p}}$. However, in the case where $T_{\rm c}/\overline{T_{\rm f}}$ was not negligible, the $\overline{T_{\rm p}}$ of the G4 could be slightly overestimated as two successive structures could be mistaken for the same structure. The extent of the relative overestimation of $\overline{T_{\rm p}}$ is less than $1+(k_{\rm f}+k_{\rm u})\alpha _i^2T_{\rm c}/2$, where $k_{\rm f}=1/\overline{T_{\rm f}}$ ( $k_u=1/\overline{T_p}$) is the real folding (unfolding) rate of the G4 structure and α_*i*_ = 0.83. This correcting factor was considered in the estimation of the errors, which are for this reason asymmetric. In the same manner, the probability that a G4 structure was formed but could not be detected was considered in the errors relative to its folding time $\overline{T_{\rm f}}$. Details methods and calculations used to determined error estimations of folding and persistence times is detailed in Supplementary Figure S8.

## RESULTS

### A single molecule assay to visualize G4 formation in real-time

**Figure 1.**
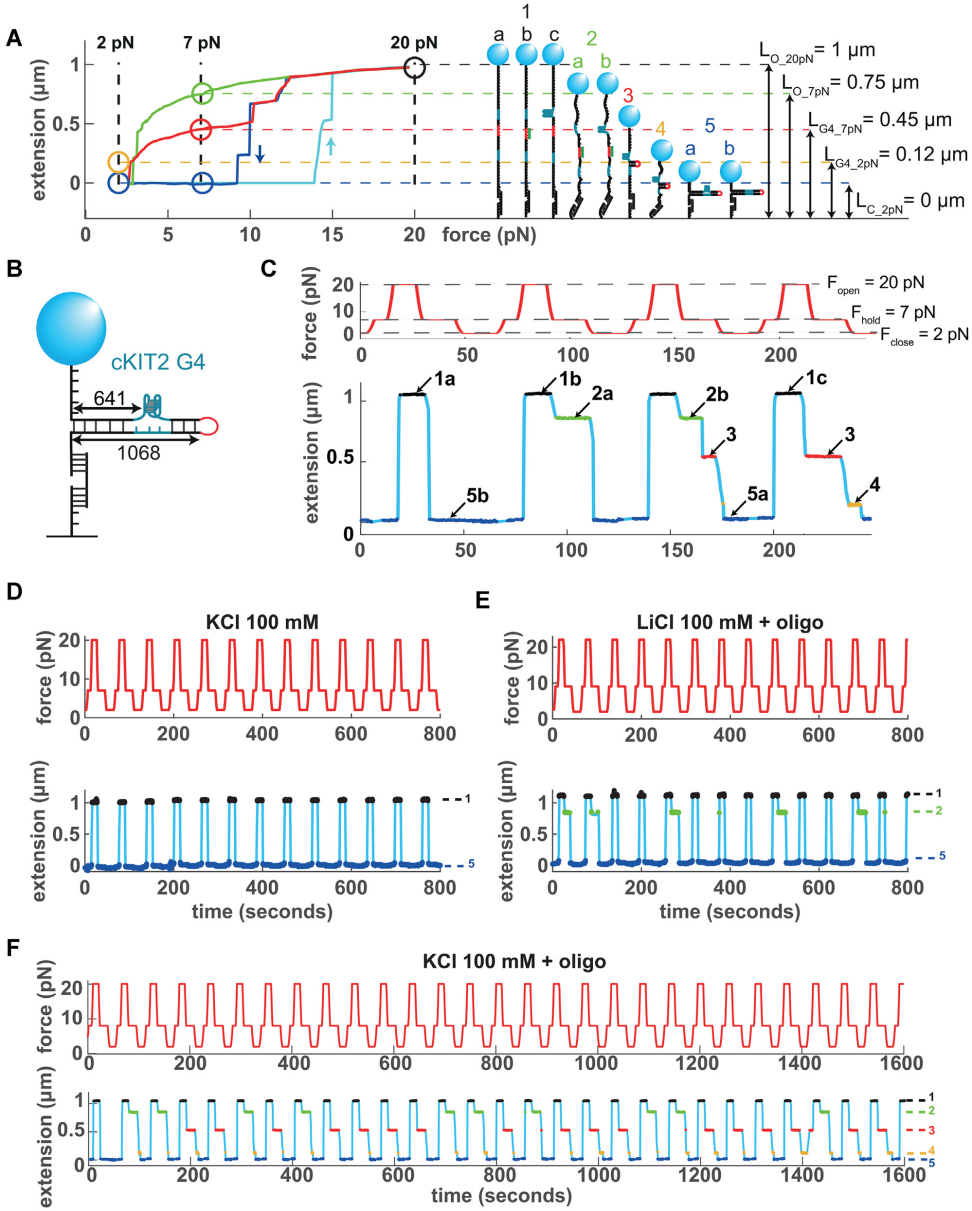
Single molecule assay for detection of G4 formation. (**A**) Left panel: Force-extension curves of a 1068 bp DNA hairpin containing a cKIT-2 G4 forming sequence. Open circles mark the different molecule extensions observed at the three test forces. Cyan: hairpin opening at increasing forces. Blue: hairpin closing with no G4 or blocking oligonucleotide at decreasing forces. Green: hairpin closing with a blocking oligonucleotide. Red: hairpin closing with a folded G4. *L*_*O* − 20 pN_: fully open hairpin at 20 *pN*. *L*_*O* − 7 pN_: oligonucleotide-blocked hairpin at 7 pN. *L*_*G*4 − 7 pN_: G4-blocked hairpin at 7 pN. *L*_*G*4 − 2 pN_: G4-blocked hairpin at 2 pN. *L*_*C* − 2 pN_: fully closed hairpin at 2 pN. Right panel: structures inferred from the force-extension curves. (**B**) Schematics of the hairpin molecule, indicating the size in bp of the full dsDNA hairpin and the position of the G4 sequence. (**C**) Force cycles (red) and extension traces (blue) illustrating the corresponding molecular states described in (A). (**D**) Force cycles (800 s, red) and corresponding extension traces (blue) of cKIT-2 hairpin in KCl only (color-coded numbers correspond to structures described in (A)). (**E**) Same as (D) in 100 mM LiCl plus blocking oligonucleotide. (**F**) Same as (D) in 100 mM KCl with blocking oligonucleotide.

To decipher G4 folding/unfolding dynamics at the molecular level, in a context where G4 structure exists in competition with a dsDNA structure, we developed a single molecule assay based on manipulation of DNA hairpin under a magnetic tweezer setup (54). Here, our molecules are ∼1.1 kb and contain one G4 forming sequence (or its non-G4 mutated version) (Figure 1A, Supplementary Figure S1, S2). In a magnetic tweezer experiment, the readout is the molecule extension, which depends on the force applied to the bead and the DNA conformation. Assuming that the formation of the G4 structure requires a single stranded intermediate to form, the assay consisted of submitting the DNA hairpin to a predetermined force cycle so as to periodically open and close the molecule. Thus, the DNA molecules alternates between single-stranded and double stranded DNA configurations. The principle of our assay is presented in Figure 1 with a G4 forming sequence from the promoter of the human cKIT oncogene (cKIT-2, supplementary Table S2) (55).

Figure 1A shows the mean force–extension trajectories of the cKIT-2 hairpin at increasing and decreasing forces. At the beginning of the experiment, starting from the situation of a closed hairpin and no force applied, when we increase the force the hairpin remains closed until the force reaches 14 pN and then the hairpin quickly opens, with a maximum extension of ∼1 μm at 20 pN (structure 1a) corresponding to a fully unzipped hairpin (cyan curve). Conversely, starting from an extended hairpin at 20 pN and decreasing the force, the hairpin refolds when the force reaches 12 pN, and is completely re-annealed at 9 pN (blue curve). However, simply opening and closing the hairpin by alternating between ssDNA and dsDNA in KCl buffer did not allow to observe G4 formation (Figure 1D).

To favor formation of the G4 structure in the ssDNA state, we added a small 7-base long oligonucleotide complementary to the hairpin loop (blocking oligonucleotide, Supplementary Table S1). If during the opened phase at *F*_open_ = 20 pN, the blocking oligonucleotide hybridized to the hairpin loop (structure 1b), it sequestered the loop, which is the hairpin nucleation spot (56). During oligonucleotide binding, the hairpin remains open until the force is reduced so much that the oligonucleotide gets ejected from the hairpin, allowing it to completely refold. In the trace shown in Figure 1A (green curve), this occurs when the force is lowered to 3 pN. The green curve therefore represents the elasticity of a 1068 bp hairpin in ssDNA state at forces from 20 to 3 pN.

Now, how do we detect the presence of a G4 structure in the hairpin? This situation is illustrated by the red curve in Figure 1A, which is observed for the cKIT-2 G4 only in the presence of KCl plus blocking oligonucleotide (Figure 1F), but not in 100 mM LiCl plus oligonucleotide condition, where G4 formation is disfavored (Figure 1E) (52). The trajectories for the red (containing a folded G4) and blue (without folded G4) curves show consistent patterns while the force is reduced from 20 to 10 pN (Figure 1A). However, in the case of the red curve, closing of the hairpin is blocked at ∼0.5 μm extension at around 10 pN. The full closing of the hairpin is delayed until the force reaches 3 pN. We attribute this difference between the red and blue curves to the formation of G4, which prevents complete closing of the hairpin in the 10–3 pN force range.

To observe the different states of the molecule, we used the force–extension trajectories to choose empirically three specific forces (Figure 1C). At *F*_open_ = 20 pN, the hairpin is fully open (∼1 μm). Since hybridization with the blocking oligonucleotide and formation of a G4 do not significantly impact on the molecule extension at 20 pN, the 1 μm extension corresponds to three indistinguishable structures: open hairpin without a bound oligonucleotide nor a folded G4 (structure 1a), open hairpin with a bound oligonucleotide (structure 1b), or open hairpin with a folded G4 (structure 1c). At *F*_hold_ = 7 pN, we observe three different extension states of the molecule: (i) oligo blockage (structure 2a or 2b, ∼0.75 μm, which corresponds to the full length molecule in ssDNA form containing or not a G4 structure), (ii) G4 blockage (structure 3, ∼0.45 μm) or (iii) no blockage (neither by a G4 nor by an oligonucleotide) (structure 5, 0 μm). Consistent with the blockage at 0.45 μm corresponding to a G4 structure, the ratio between the two extensions at 7 pN (0.45/0.75) represents 60$\%$ of the total extension, which for this cKIT-2 hairpin of 1068 bp long corresponds exactly to the distance between the G4 sequence and the ssDNA–dsDNA junction (0.6 × 1068 bp = 641 bp) (Figure 1B). At *F*_close_ = 2 pN, the hairpin is fully closed (structure 5a or 5b, 0 μm, which corresponds to a dsDNA hairpin containing or not a folded G4). In addition, only in the cases where a G4 structure is formed, a 0.12 μm extension state can be observed at 2 pN, which can be attributed to a relaxed form of structure 3 (Figure 1A, C, F, structure 4).

Practically, the assay consisted on imposing the three forces successively on the molecule during multiple cycles (Figure 1C–F). Figure 1C illustrates the extensions corresponding to different molecular states detected during force cycles. Evidence of the persistence of the G4 throughout multiple cycles is then provided by successive blockage at 0.45 μm. These data would suggest that the G4 gets embedded in a dsDNA bubble during the phase at 2 pN (structure 5a) and resists to the pulling force at 20 pN (structure 1c). Different scenarios to determine the persistence of a G4 from extension traces is described in Supplementary Figure S9.

### Folding of G4 structures from human telomeres, human oncogene promoters and an avian replication origin

**Figure 2.**
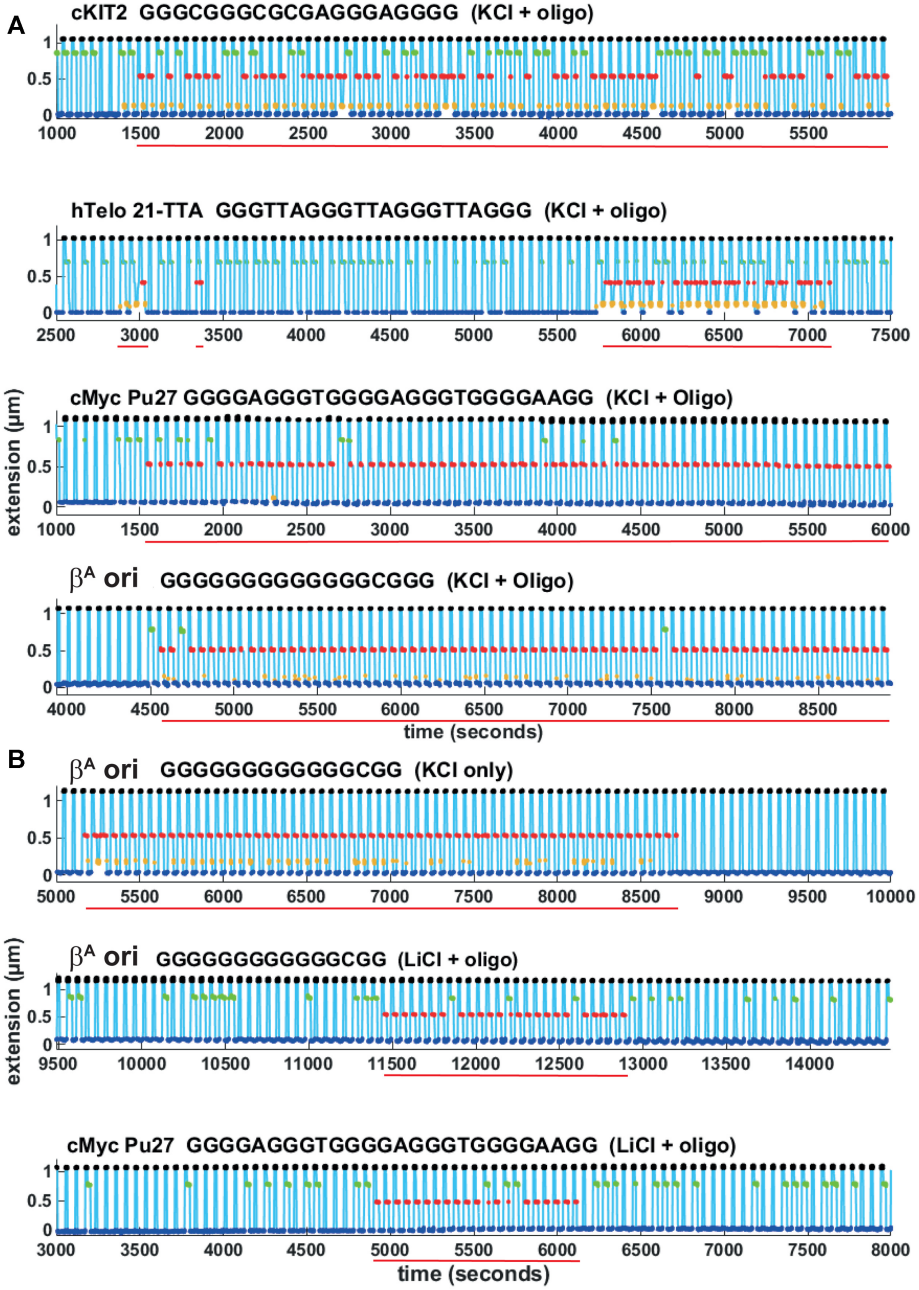
Extension traces from single molecules containing cKIT-2, hTelo 21-TTA, cMYC-Pu27 or ori β^*A*^ G4 forming sequences. (**A**) From top to bottom, cKIT-2, hTelo 21-TTA, cMYC and ori β^*A*^ G4 forming sequences in 100 *mM* KCl and blocking oligonucleotide. (**B**) ori β^*A*^ G4 forming sequence in KCl without blocking oligonucleotide (top) and in LiCl plus blocking oligonucleotide (middle). cMYC-Pu27 in LiCl plus oligonucleotide (bottom). Formation of a G4 structure is detected by a blockage at 7 pN (red), as described in Figure 1. Single G4 events are underlined (red).

We used this assay to compare the behavior of several well studied G4 forming sequences in our assay in addition to cKIT-2, namely human telomeric repeats (hTelo 21-TTA) (57), the promoter of the human oncogene cMYC (cMYC-Pu27) (58), and a replication origin found in the chicken genome (ori β^*A*^) (10). We performed experiments lasting up to 16 hours representing several thousands force cycles. Figure 2A shows 5000 s long snapshots of extension traces obtained with the four sequences, in presence of 100 mM KCl and blocking oligonucleotide. Formation of G4 structures could be observed for all sequences on most tested beads, demonstrating the generality of the assay to study formation of intramolecular G4s. Recordings and automatic detection of molecule states from the full course of representative experiments can be found in Supplementary Figure S3–S5. Experiments were also performed in control conditions (100 mM LiCl with or without blocking oligonucleotide, or 100 mM KCl without blocking oligonucleotide). No blockage corresponding to G4 structures was observed in either control conditions for cKIT-2 and hTelo 21-TTA. We could, however, detect formation of G4 structures, while less frequent and short-lived, in 100 mM LiCl plus blocking oligonucleotide for cMYC-Pu27 and ori β^*A*^ sequences (Figure 2B and Supplementary Table S3). We measured circular dichroism spectra of oligonucleotides containing the cMYC-Pu27 and ori β^*A*^ sequences in LiCl buffer, which exhibited the characteristic profile of parallel G4 structures (Supplementary Figure S6). The ori β^*A*^ sequence also exhibited G4 formation in 100 mM KCl without blocking oligonucleotide (Figure 2B and Supplementary Figure S5). Therefore, our assay can be used to characterize unambiguously rare G4 folding events.

### Comparative folding and unfolding dynamics of intramolecular G4s

Next, we measured the folding and persistence times of the G4 structures in our assay. To gain insights into the folding and unfolding dynamics, we recorded multiple events of G4 formation over several beads for each G4 forming sequence (recording more than a hundred G4 events per sequence, each experiment lasting up to 16 h). We were able to monitor a field of multiple molecules at the same time to obtain statistically significant measurements of the G4 folding and persistence times under various conditions. For each sequence studied, we compiled the experimental folding times (*T*_f_) as all the times where the blocking oligonucleotide delayed hairpin refolding at *F*_hold_ (extension of 0.75 μm at 7 pN) before observing G4 folding (extension of 0.45 μm at 7 pN). Similarly, we defined persistence times (*T*_p_) as the time spent by a molecule in a G4 conformation before the structure unfolded. The average folding time ($\overline{T_{\rm f}}$) of a given sequence was therefore obtained by dividing the sum of *T*_f_ over all beads and over the entire acquisition, by the number of times distinct G4 events were observed. Thus, the inverse of $\overline{T_{\rm f}}$ ($k_{\rm f}=1/\overline{T_{\rm f}}$) is a direct measure of the probability of folding a G4 structure per unit of time spent at 7 *p*N. A longer average folding time indicates a smaller folding probability while a shorter average folding time indicates a larger folding probability. Briefly, the average persistence time ($\overline{T_{\rm p}}$) is acquired by dividing the sum of *T*_*p*_ over all beads and over the entire acquisition, by the number of times a G4 is unfolded (corresponding to the first absence of blockage at both 0.75 and 0.45 μm extension following detection of a G4 structure). Therefore $\overline{T_{\rm p}}$ represents the stability of a given G4 structure in a context alternating between ssDNA and dsDNA. The inverse of $\overline{T_p}$ ($k_{\rm u}=1/\overline{T_{\rm p}}$) indicates the unfolding rate of the G4 structure. Details on $\overline{T_{\rm f}}$ and $\overline{T_{\rm p}}$ calculation is given Supplementary Figure S7.

Our assay involves a periodic testing of the presence of a G4 in the substrate at every cycle. During a fraction of this cycle, the presence of the G4 cannot be detected, either because the hairpin is open, because the G4 is embedded in double-stranded DNA or the hairpin is blocked by an oligonucleotide. Thus, there is a probability that a G4 structure unfolds and then refolds during this time. We could then in theory observe two successive blockages at the G4 position that we would consider as a unique structure while in reality, two successive folding and unfolding events happened. This probability depends on the dynamic of the structure and can be calculated. Details on calculation of this probability are given in Supplementary Figure S8. This probability is low for most of G4 studied here except for ori β^*A*^. However, this probability was taken into account in the error estimation of $\overline{T_{\rm f}}$ and $\overline{T_{\rm p}}$.

**Figure 3.**
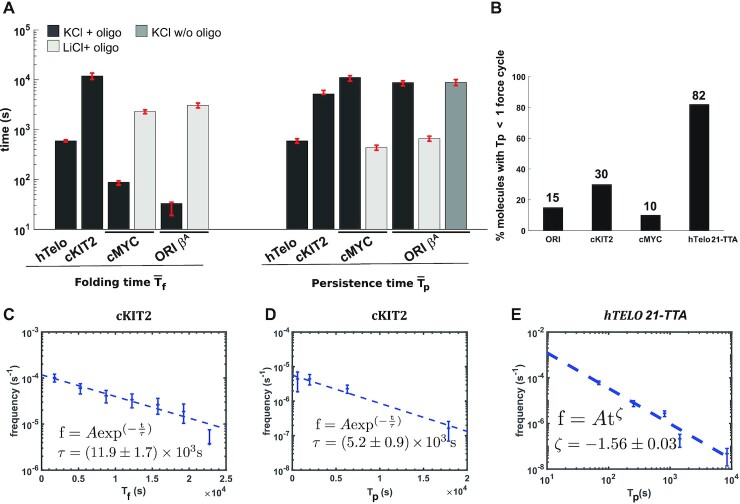
Folding time and persistence time of G4 structures. (**A**) Average folding times and persistence times of G4 structures. (**B**) Percentage of G4 structures with a persistence time shorter than one force cycle. Frequency of folding times (**C**) and persistence times (**D**) of cKIT-2 G4. Both are fitted with a first order exponential law. (**E**). Frequency of persistence time of hTelo 21-TTA G4 and power law fit of the data.

The dynamic properties of all G4 structures tested in this work are shown in Table 1 and Figure 3A. The four studied G4 sequences (hTelo 21-TTA, cKIT-2, cMYC-Pu27 and ori β^*A*^) displayed large difference on their $\overline{T_{\rm f}}$ (from ∼30 to ∼12 000 s) and $\overline{T_{\rm p}}$ (from ∼500 to ∼11 000 s) at 25°C in 100 mM KCl plus blocking oligonucleotide. Some G4 sequences, such as cMYC-Pu27 and ori β^*A*^, had a very short folding time and very long persistence time. In comparison, cKIT-2 exhibited a longer folding time and a shorter persistence time compared to cMYC-Pu27 and ori β^*A*^. Finally, hTelo 21-TTA presented the most dynamic structures, with relatively short folding and short persistence times.

**Table 1.** Average folding and persistence times of G4 structures in different conditions

	Conditions	cMYC	cKIT-2	ori β^*A*^ wt	ori β^*A*^ m12	ori β^*A*^ m16	21-TTA	45-TTA	21-CTA
	LiCl	nd	nd	–	nd	nd	nd	nd	nd
$\overline{T_{\rm f}}$ (s)	LiCl + oligo	2300 ± 200	–	3000 ± 350	–	–	–	–	–
	KCl	–	–	nd	–	–	–	–	
	KCl + oligo	90 ± 10	12 000 ± 1700	30 ± 13	1400 ± 70	9000 ± 1300	350 ± 30	150 ± 13	1700 ± 130
	LiCl	nd	nd	–	nd	nd	nd	nd	nd
$\overline{T_{\rm p}}$ (s)	LiCl + oligo	400 ± 50	–	650 ± 8	–	–	–	–	–
	KCl	–	–	9000 ± 1200	–	–	–	–	–
	KCl + oligo	11 000 ± 1700	5000 ± 900	9000 ± 1300	6500 ± 1300	125 ± 20	600 ± 80	500 ± 50	350 ± 60

nd: not determined. –: no G4 formation observed. means ± estimation error are indicated.

The distribution of folding (*T*_f_) and persistence (*T*_p_) times followed in most of the cases an exponential law, as exemplified for the cKIT-2 G4 in Figure 3C and D. This suggests folding and unfolding of the G4 in a single step. However the persistence time of the hTelo 21-TTA G4 is best fitted by a power law (Figure 3E), which would indicate a mixture of indistinguishable conformations with different ($\overline{T_{\rm p}}$), corroborating previous results pointing to structural diversity of human telomeric G4 (43).

By design, one force cycle corresponds to the lower-resolution limit of our assay. Therefore, structures that were stable less than one cycle could represent unstable structures either with a short persistence time or structures that are mechanically unfolded during the pulling step at 20 pN. These ambiguous events were thus not considered in the calculation of $\overline{T_{\rm p}}$. Such unstable structures were frequently observed for the telomeric G4 (21-TTA), with $82\%$ of the structures observed exhibiting a persistence time shorter than one cycle. For cMYC-Pu27, cKIT-2 and ori β^*A*^ G4s, the structures lasted less than one cycle represented respectively $10\%, 30\%$ and $15\%$ of the events observed (Figure 3B). Interestingly, as noted above, we could also observe for the ori β^*A*^ and the cMYC-Pu27 G4s a blockage at 0.45 μm extension in LiCl in presence of a blocking oligonucleotide (Figure 3 and Supplementary Figure S4 and S5). However, the average folding time of the structures was longer (∼26-fold increase for cMYC-Pu27 and 100-fold increase for ori β^*A*^), and their average persistence time was significantly shorter (respectively 27-fold and 14-fold for cMYC-Pu27 and ori β^*A*^) than the ones obtained in KCl plus blocking oligonucleotide. The lower persistence of the structures observed in LiCl compared to KCl confirms that the blockage at 0.45 μm is due to G4 structures. We cannot completely rule out the contribution of a structure forming on the opposite C-rich strand, namely an i-motif, although the neutral pH buffer conditions used in our assay should disfavor its formation (58). In the absence of the blocking oligonucleotide and in 100 mM KCl, ori β^*A*^ could form a structure with similar persistence (9000 s) than the one in KCl plus oligonucleotide. This result suggests two additional possibilities: the ori β^*A*^ sequence might not require the opening of dsDNA to form a G4 or it may fold in a ssDNA context even at high force (20 pN).

### Folding time and persistence time of ori β^*A*^ mutants

Sequences composed of long guanine stretches (such as the ori β^*A*^ sequence) form preferentially intermolecular G4s in bulk experiments, preventing from assessing the effect of intramolecular G4 folding. Our single-molecule assay helps to overcome this issue. In order to test the sensitivity of the ori β^*A*^ sequence to mutation in our assay, we measured the dynamics of mutated sequences of ori β^*A*^ containing an adenine instead of a guanine at position 12 (m12) or at position 16 (m16) (Table 1 and Supplementary Figure S10C). These point mutations were previously reported to lower the ability of these sequences to form G4 structures based on prediction from sequence features and were shown to correlate with lower replication origin activity *in vivo* (10). In our assay, the two ori β^*A*^ mutants could still fold into a G4 structure, although they exhibited a much longer folding time compared to wild-type (wt) ori β^*A*^ (46-fold and 300-fold increase for m12 and m16 respectively). The m12 G4 structure exhibited a long persistence time, similar to the wt ori β^*A*^ structure, while m16 G4 had a much shorter $\overline{T_{\rm p}}$ (72-fold) compared to wt and to m12. In addition, m16 presented ∼4.5-fold increase in the percentage of G4 structures with very short persistence time (≤1 force cycle) compared to wt and to m12 ($69 \%$ for m16 instead of $15\%$ for wt and m12) (Supplementary Figure S10B). These results show that our assay can reveal intramolecular G4 dynamics and the effect of subtle sequence difference in G4 forming sequences made of long guanine stretches. In the case of the ori β^*A*^ mutants, we see that the guanine at position 12 only affects the folding rate of the G4, whereas the guanine at position 16 is critical for the efficient folding and persistence of the G4 structure in the dsDNA context. These results are consistent with G4 folding prediction made from the ori β^*A*^ sequence. The wt ori β^*A*^ sequence has the possibility to form at least ten different three-quartet G4 structures. However, the m12 mutant conserves only one possibility out of ten to form a three-quartet G4, which could explain its lower folding rate compared to the wt sequence. The m16 mutant can form two-quartet G4s or a three-quartet G4 with zero nucleotide-loops, which affects both folding and unfolding rates.

### Folding time and persistence time of dimeric human telomeric repeats and of the common CTAGGG variant

Human telomeres contain variant repeats interspersed with the consensus TTAGGG motif. The CTAGGG variant, when present as a contiguous array within the telomere, causes telomere instability in the male germ line and somatic cell (59). It has been shown that four successive CTAGGG repeats were prone to form a G4 *in vitro*, and adopted a different G4 conformation than the wt (TTAGGG)_4_ repeats in KCl (60). In our assay, the formation of the 21-CTA variant in KCl was five times slower than the wt 21-TTA G4 ($\overline{T_{\rm f}}=1700$ s versus 350 s). The persistence time of the variant 21-CTA G4 was also reduced by ∼2-fold compared to the wild type ($\overline{T_{\rm p}}= 350\,{\rm s}$ versus 600 s) (Supplementary Figure S11A, B and Table 1). Therefore, the variant cytosine affects the folding ability of the G4 and has a modest effect on its persistence time. Altogether, these results indicate that the CTAGGG variant has a slightly lower ability to fold into a G4 and to persist in DNA than the wt telomere repeat, thus suggesting that the increase of genomic instability at human telomeres due to this variant can not be accounted by G4 formation on its own.

Since telomeres are composed of tandem repeats *in vivo*, we also measured the folding and unfolding dynamics of one versus two G4 units (45-TTA) (Supplementary Figure S11A, B and Table 1). Folding of this sequence into a G4 took in average half the time ($\overline{T_{\rm f}}$ = 150 s) compared to the 21-TTA sequence in KCl. This difference in mean folding time between one and two G4 units is compatible with independent folding of the two G4 structures, suggesting no or little interaction of the tandem G4s in our system. The 45-TTA G4 displayed a persistence time ($\overline{T_{\rm p}}$ = 500 s) similar to the one of 21-TTA G4 ($\overline{T_p}$ = 600 s), indicating again a lack of a cooperative effect on the persistence time of the structures. The decrease in folding times suggests a higher probability to form the G4 structure in the case of the repeated sequence 45-TTA. This is compatible with the larger number of G4 nucleation sites permitted by the repeat of G4 motifs (Supplementary Figure S11C). Among these five nucleation sites, three of them (position 2/3/4) prevent the formation of two consecutive G4 structures. Thus, the probability that the 45-TTA sequence folds into a unique G4 structure is higher than the probability to form into two consecutive ones. However, our assay does not allow distinguishing effectively between the formation of one or two structures in this case.

### G4 stabilizing ligands affect both G4 folding and unfolding dynamics

Finally, we wanted to assess the effect of G4 ligands on the kinetics of G4 folding and persistence in our assay. The potential to manipulate G4 formation at telomeres or oncogene promoters have made them an attractive target for antitumoral drug design (61–64). Small molecules and antibodies have been developed for targeting G4 *in vitro* and have been widely used to evidence G4 formation *in vivo* and potentialize their biological effect (47,65,66). We tested how such molecules affect folding and unfolding dynamics of G4 structures in the context of our single molecule assay, using the 21-CTA telomere variant as a model sequence (Table 2). As demonstrated above, this sequence has the benefit of exhibiting a long folding time ($\overline{T_{\rm f}}$ = 1700 s) and a short persistence time ($\overline{T_{\rm p}}$ = 350 s), therefore allowing any effect of the ligand on these two values to be easily measurable.

**Table 2.** $\overline{T_{\rm f}}$ and $\overline{T_{\rm p}}$ of hTelo 21-CTA at different concentration of ligand 360B or BG4 antibody

[360B]	100 nM	500 nM	1 μM	1.5 μM	2 μM
$\overline{T_{\rm f}}$ (s)	1350±110	750±110	300±35	130±15	70±10
$\overline{T_{\rm p}}$ (s)	300±30	450±60	350±50	1050±250	1350±300
[BG4]	20 nM	100 nM	200 nM		
$\overline{T_{\rm f}}$ (s)	1050±130	400±30	150±15		
$\overline{T_{\rm p}}$ (s)	300±70	1150±150	1250±250		

Means ± estimation error are indicated.

**Figure 4.**
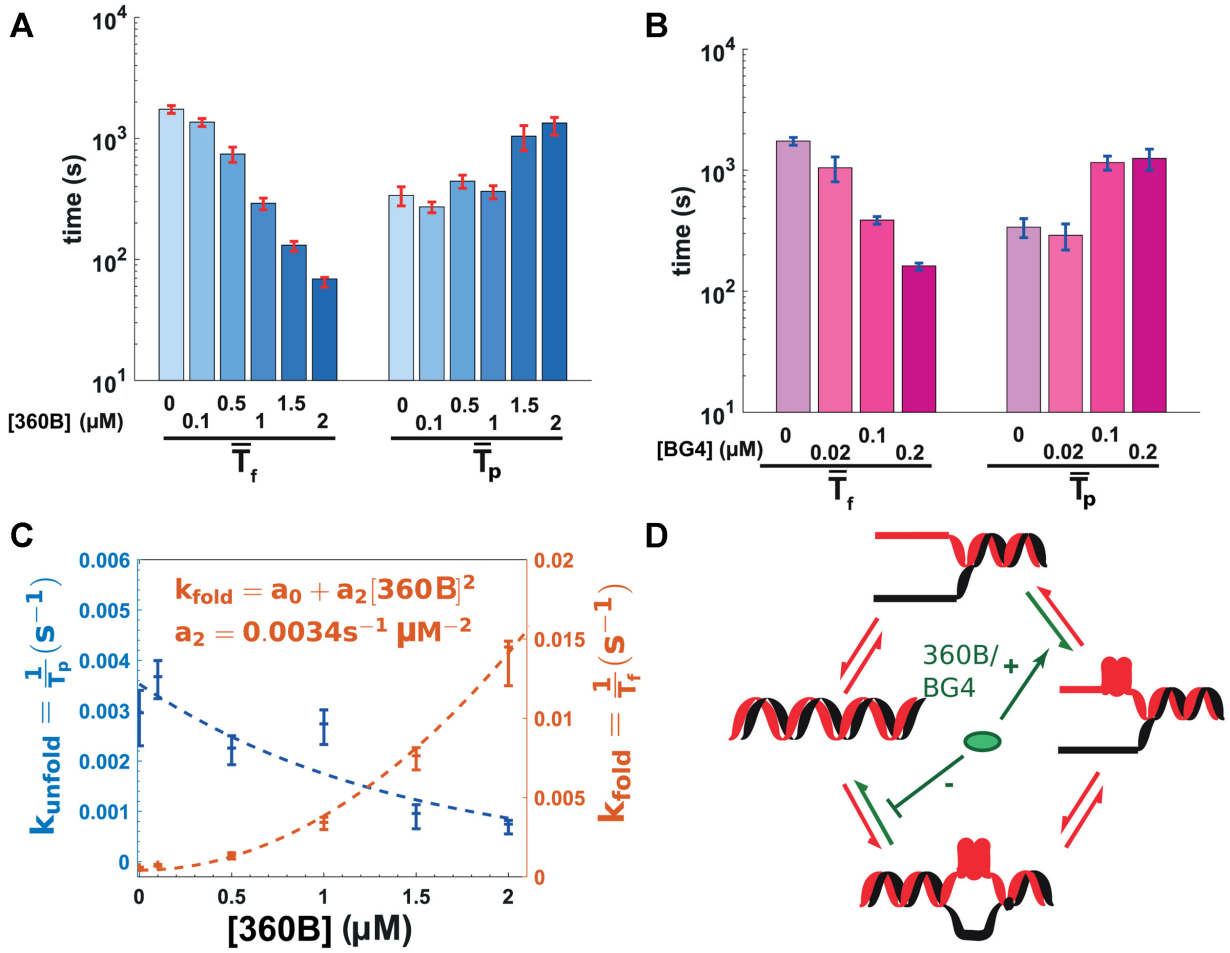
Effect of G4 ligand 360B and BG4 antibody on G4 folding and persistence times. (**A**) Folding and persistence times of 21-CTA telomeric G4 with increasing concentration of the small ligand 360B. (**B**) Folding and persistence times of 21-CTA G4 with increasing concentration of BG4 antibody. (**C**) Folding and unfolding rates of the 21-CTA G4. Fitting equations of the experimental data are displayed. (**D**) Schematic diagram showing the effect of a G4 binder on both folding and unfolding rates of G4 embedded in dsDNA. G4 ligand and antibody (green ellipse) may favor the folding as well as the persistence of G4.

We investigated the effect of 360B, a selective G4 ligand of the pyridine derivative series, on both folding and unfolding dynamics of the CTA variant G4. The pyridine dicarboxamide derivative 360B is a more water-soluble formulation of the commonly used ligand 360A (Supplementary Figure S12A) (46,67). We measured the folding and persistence times of the G4 in 100 mM KCl and blocking oligonucleotides with increasing concentrations of 360B (Figure 4). First, we observed that the percentage of structures lasting less than one open/close cycle decreases from $88\%$ to $45\%$ when the ligand concentration increases from 0 to 2 μM, confirming the stabilization of the structures (Supplementary Figure S12B). Second, increasing concentration of 360B affected both the folding time and the persistence time of the structure in a dose-dependent manner. With increasing concentrations of 360B, we observed higher G4 folding rate (decreased $\overline{T_{\rm f}}$) and more persistent G4s (increased $\overline{T_{\rm p}}$), suggesting that the ligand favors the formation of the structure and increases its persistence time (Figure 4A). Interestingly, $k_{{\rm fold}} = 1/\overline{T_{\rm f}}$ increased non-linearly with the concentration of ligand (Figure 4C). The data fit to a quadratic polynomial equation (*R*^2^ = 0.9957), which would suggest that two ligand molecules bind simultaneously on one G4 structure. This is reminiscent of what has been reported previously with the interaction between human telomeric G4 and the ligand CuII-tolyterpyridine (68). The ligand also affected the persistence time of the structure. Indeed, *k*_u_ = $1/\overline{T_{\rm p}}$ decreased with increasing concentration of 360B, with the data best fitted by a first order exponential decay. We also tested the effect of 360B on the cKIT-2 G4, a structure which exhibits long $\overline{T_{\rm f}}$ and long $\overline{T_{\rm p}}$. Similarly to the 21-CTA G4, we observed a higher folding rate and a longer persistence time of the cKIT-2 G4 in the presence of ligand (Supplementary Figure S13). 100 nM of 360B was sufficient to reduce $\overline{T_{\rm f}}$ by 4-fold and increase $\overline{T_{\rm p}}$ by almost 20-fold. Because of its already long persistence time, most of the cKIT-2 G4 ($\sim 60\%$) were not unfolded during the recorded time of the experiment (≥90 000 s) at a concentration of 100 nM of 360B.

Finally, we tested the effect of the anti-G4 single chain BG4 antibody (47) on the 21-CTA sequence. Similarly to our results with 360B, BG4 elicited a dose-dependent effect on both *T*_f_ and *T*_p_ (Figure 4B and Supplementary Figure S12). Therefore, the two ligands behave in our assay by favoring G4 formation in ssDNA and slowing down its unfolding, as schematized in Figure 4D.

## DISCUSSION

In this work, using a single molecule DNA manipulation assay, we compared the dynamics of G4 folding and unfolding for several G4 forming sequences in a context where ssDNA exists in equilibrium with dsDNA. Here, we studied well-known G4 motifs, including human telomeres, human oncogene promoters of cMYC and cKIT genes, and a chicken replication origin.

Overall, our results are in agreement with previously determined parameters from bulk studies (Table 3). However, our assay allows accessing folding and persistent rates, that are likely to be more reflective of their dynamic *in vivo*. Indeed, determining both folding and unfolding rates leads to interesting observations even for stable G4s. For example, although the cKIT-2 G4 exhibits a high melting temperature (*T*_m_ = 70°C), our assay reveals that it has a low folding rate. In comparison, the telomeric G4 hTelo 21-TTA exhibits a similar *T*_m_ of 68°C but shows much faster dynamics. Our textbook view of G4 formation in genomic DNA is that the folding of such structure needs a transient ssDNA intermediate, which could likely occur during replication, transcription or DNA repair (71). Indeed, to form in our assay, most structures required a step in a single strand conformation at low force. Interestingly, in the case of the ori β^*A*^ sequence, G4 can form in absence of this step at low force. This suggest that ori β^*A*^ can form a G4 in ssDNA at high force, or that it can form spontaneously in dsDNA. In addition, this is the first time that an intramolecular G4 formed by a long guanine stretches like the ori β^*A*^ sequence was experimentally observed, since they tend to fold into intermolecular G4 structures (or a mixture of intra- and intermolecular G4s) at the DNA concentrations required for common *in vitro* spectroscopic studies (10). Finally, although our label-free assay is a simplistic model with regard to an actual *in vivo* situation, the observed dynamics of the G4 structures are consistent with their purported role *in vivo*. In the case of a replication origin G4, fast folding and long persistence time in a dsDNA should be advantageous if the structures serve as anchors for protein complexes prior to replication licensing as previously proposed (38). Although not forming as easily as the ori β^*A*^ G4, the two promoter G4s, cMYC and cKIT, also displayed long persistence in dsDNA, which is consistent with a sustained effect on transcription activity (72).

**Table 3.** Comparison of $\overline{T_{\rm p}}$ and $\overline{T_{\rm f}}$ with melting temperatures (*T*_m_) and unfolding forces determined in identical buffer conditions (10 mM Tris–HCl pH 7.5, 100 mM KCl), ranked by decreasing *T*_m_)

G4	$\overline{T_{\rm p}}$ ± error	$\overline{T_{\rm f}}$ ± error	*T*_p_ ≤ *T*_c_	*T*_m_ (°C)	Unfolding force (pN)
β^*A*^ ori	9000±1300	30±13	15$\%$	80 (10)	nd
cMYC	11 000±1700	90±10	10$\%$	78 (69)	22, 50 (42)
cKIT-2	5000±900	12 000±1700	30%	70 (70)	nd
21-TTA	600±80	350±30	82%	68 (68)	11, 22 29, 36 (43)
21-CTA	350±60	1700±130	88%	67 (70)	nd
45-TTA	500±5	150±13	83%	59 (70)	nd
β^*A*^-m12	6500±1300	1400±170	15%	55 (10)	nd
β^*A*^-m16	125±20	9000±1300	69%	45 (10)	nd

nd: not determined. *T*_c_: cycle time. references in parentheses.

At the other end of the spectrum, human telomeric sequences were the weakest of the tested structures. They were rapid to form, but showed limited mechanical stability under a 20 pN force and exhibited relatively short persistence time, compared to other sequences. The short persistence (less than one force cycle) observed with human telomeric G4s (Supplementary Figure S11) can be attributed either to a low mechanical stability at high force (20 pN) or to a low persistence within a dsDNA bubble. Although we cannot distinguish between these two possibilities, previous single-molecule assays have shown that the human telomeric G4 repeat could fold into four different G4 conformations, two of them being unstable around 20 pN (Table 3). The distribution of persistence times measured with our assay was best fitted by a power law, also suggesting the contribution of several structures. Our results corroborate other former single molecule studies (43) and point out that a single human telomeric G4 is a dynamic structure, forming promptly in ssDNA but also dissolved rapidly under mechanical forces or in the presence of a complementary strand. In the human genome, the telomeric sequence TTAGGG is mainly embedded in a large tandemly repeated dsDNA regions which may form multiple consecutive G4s. This raises the possibility that the impediment to molecular motors when progressing through telomeric repeats owes little to the stability of individual G4 structures alone, at least in the dsDNA portion of telomeres, but probably to other factors, like DNA-bound proteins.

Our results using G4 stabilizing ligands showed that the 360B ligand and the BG4 antibody significantly increased the formation rate of the G4s, as well as their persistence time, proving that they favor G4 formation in addition to stabilizing the structure, as previously noted (66,73). In this regard, our assay could help characterize ligands or antibodies which perturb less (or more) folding rates and would be a useful addition for *in vivo* physiological studies of G4 dynamics.

## CONCLUSION

Contrarily to previous single-molecule measurements that were performed at constant forces and without the presence of a complementary strand (39–45), our assay allows *in vitro* measurement of G4 folding and persistence times in a context that simulates the competitive environment of G4 structures formation *in vivo*. This assay could be easily adapted to study various biochemical processes (in first place replication and transcription), by tuning the parameters of the force cycles and by studying the effect of proteins involved in G4 processing. It is noteworthy that such modifications in the force cycles significantly affect the kinetics of formation but not the persistence time of a G4 structure (Supplementary Figure S14). Therefore, our assay allows assessing a wide range of G4 folding dynamics while the DNA molecule is subjected to different mechanical stresses or G4-interacting ligands. Finally, gathering folding rates and persistence times of a wider array of sequences in this assay could help improve the predictive power of computational methods developed to find G4 forming sequence in genomic data and score their ability to form a stable structure in a dsDNA context (74).

## DATA AVAILABILITY

The data that support the findings of this study are available on request from the corresponding authors.

## Supplementary Material

gkab306_Supplemental_File
